# A nation-wide multicenter 10-year (1999–2008) retrospective study of chemotherapy in Chinese breast cancer patients

**DOI:** 10.18632/oncotarget.16439

**Published:** 2017-03-22

**Authors:** Qiao Li, Zhao Yang, Jinhu Fan, Jianjun He, Bin Zhang, Hongjian Yang, Xiaoming Xie, Zhonghua Tang, Hui Li, Youlin Qiao, Pin Zhang

**Affiliations:** ^1^ Department of Medical Oncology, National Cancer Center/Cancer Hospital, Chinese Academy of Medical Sciences (CAMS) and Peking Union Medical College, Beijing, China; ^2^ Department of Cancer Epidemiology, National Cancer Center/Cancer Hospital, Chinese Academy of Medical Sciences (CAMS) and Peking Union Medical College, Beijing, China; ^3^ Department of Oncology Surgery, First Affiliated Hospital, School of Medicine of Xi’an Jiaotong University, Xi'an, China; ^4^ Department of Breast Surgery, Liaoning Cancer Hospital, Shenyang, China; ^5^ Department of Breast Surgery, Zhejiang Cancer Hospital, Hangzhou, China; ^6^ Department of Breast Oncology, Sun Yat-Sen University Cancer Center, Guangdzhou, China; ^7^ Department of Breast–Thyroid Surgery, Xiangya Sencod Hospital, Central South University, Changsha, China; ^8^ Department of Breast Surgery, The Second People's Hospital of Sichuan Province, Chengdu, China

**Keywords:** transgelin2, SREBP, PDAC, diabetes, insulin

## Abstract

Little information is available on the evolvement of chemotherapeutic regimens administered to Chinese females with breast cancer. We retrospectively analyzed demographic, pathological and chemotherapeutic data of 4211 breast cancer patients, who were randomly selected from representative hospitals of 7 traditional areas in China between 1999 and 2008. A total of 3271 cases (77.7%) received adjuvant chemotherapy, 558 (13.3%) received neoadjuvant chemotherapy, and 392 (9.3%) received chemotherapy for metastatic disease. In the adjuvant setting, higher percentage of patients with younger age, advanced stage, hormone receptor (HR) negative or HER2 positive disease received chemotherapy (P<0.001). The use of CMF (cyclophosphamide, methotrexate and 5-fluorouracil) in adjuvant chemotherapy decreased significantly from 1999 to 2008, while the use of anthracycline-based (without taxanes) regimens increased in the first 5 years, followed by increased use of regimens containing both anthracyclines and taxanes. Women with locally advanced disease received more neoadjuvant chemotherapy. The percentage of neoadjuvant regimens containing anthracyclines and taxanes increased during this period. In first-line chemotherapy of metastatic disease, 87.5% of cases received combined chemotherapy, and platinum-based regimens were also major choices aside from anthracyclines and taxanes. In second-line chemotherapy, 80.3% received combined chemotherapy, and the combination of taxane and platinum was the most common choice. In conclusion, major changes have taken place in breast cancer chemotherapy in China during this 10-year interval, which reflected the incorporation of key evidence and guidelines into Chinese medical practice.

## INTRODUCTION

Chemotherapy of breast cancer is a rapidly evolving field. In the last quarter of the previous century, the combination of CMF was demonstrated to reduce risks of recurrence and death in early breast cancer (EBC) women [1]. In the 1980s, anthracycline-based combinations were proved effective in the adjuvant setting, followed by taxanes in the 1990s [2–4]. There are agents such as capecitabine, vinorelbine, gemcitabine, or other targeted therapies that have been approved for the treatment of metastatic breast cancer (MBC) [5]. In addition, neoadjuvant chemotherapy is increasingly applied due to higher breast conserving rates and the study of novel agents and regimens [6].

At present, little was known about the evolvement and influencing factors of breast cancer chemotherapy in China. In the current analysis, we attempted to investigate the use of chemotherapeutic regimens and agents for 4211 patients selected from representative hospitals of 7 traditional regions in China between 1999 and 2008. Additionally, this study was also aimed to improve understanding about incorporation of new evidence and guidelines into Chinese practice. Due to possible selection bias of representative hospitals, the data collected in our study may not be completely identical with the reality of China.

## RESULTS

A total of 4211 patients in the 7 geographically representative hospitals from 1999 to 2008 were randomly selected in this analysis. As demonstrated in Table 1, 3271 cases (77.7%) received adjuvant chemotherapy, 558 (13.3%) received neoadjuvant chemotherapy, and 392 (9.3%) received chemotherapy for MBC. The percentage of patients who received neoadjuvant chemotherapy gradually increased from 10.4% in 1999 to 16.1% in 2008, while application of chemotherapy in adjuvant and metastatic settings remained relatively stable during this 10-year period. Comparing different regions (Table 2), the percentages of adjuvant and neoadjuvant chemotherapy were the highest in the southwest area (91.8% and 26.4%, respectively) and were the lowest in the northwest (57.6% and 1.5%, respectively). The percentage of chemotherapy in metastatic setting was the highest in the south area (18.1%) and was the lowest in the central (2.0%).

**Table 1:** Frequency distribution of chemotherapy in different settings from 1999 to 2008

	1999		2000		2001		2002		2003		2004		2005		2006		2007		2008		Total	
	n	%	n	%	n	%	n	%	n	%	n	%	n	%	n	%	n	%	n	%	n	%
No chemotherapy	78	19.35	64	18.29	71	18.83	48	14.08	53	13.59	49	11.75	38	9.36	62	13.42	96	16.9	67	13.48	626	14.87
Neoadjuvant	42	10.42	29	8.29	34	9.02	51	14.96	37	9.49	82	19.66	38	9.36	87	18.83	78	13.73	80	16.1	558	13.25
Adjuvant	299	74.19	265	75.71	290	76.79	270	79.18	305	78.21	335	80.34	333	82.02	362	78.35	431	75.88	381	76.66	3271	77.68
Metastatic	35	8.68	35	10	32	8.49	31	9.09	47	12.05	37	8.87	44	10.84	35	7.58	62	10.92	34	6.84	392	9.31
Unknown	14	3.47	13	3.71	8	2.12	8	2.35	13	3.33	9	2.16	19	4.68	25	5.41	15	2.64	33	6.64	157	3.73
Total	403		350		377		341		390		417		406		462		568		497		4211	

**Table 2:** Frequency distribution of chemotherapy in different settings in 7 geographic regions

	North		East		South		Northeast		Central		Northwest		Southwest		Total	
	n	%	n	%	n	%	n	%	n	%	n	%	n	%	n	%
No chemotherapy	155	24.18	27	4.46	75	12.42	113	13.58	37	6.78	197	40.79	22	4.41	626	14.87
Neoadjuvant	43	6.71	118	19.47	61	10.1	180	21.63	18	3.3	7	1.45	131	26.25	558	13.25
Adjuvant	390	60.84	518	85.48	484	80.13	645	77.52	498	91.21	278	57.56	458	91.78	3271	77.68
Metastatic	34	5.3	99	16.34	109	18.05	61	7.33	11	2.01	55	11.39	23	4.61	392	9.31
Unknown	88	13.73	35	5.78	7	1.16	18	2.16	9	1.65	0	0	0	0	157	3.73
Total	641		606		604		832		546		483		499		4211	

### Adjuvant chemotherapy

#### Factors affecting adjuvant chemotherapy selection

Logistic regression analysis was performed to evaluate factors that might affect the application of adjuvant chemotherapy (Table 3). Further multivariate analysis demonstrated that age, stage, HR and HER2 status were independent predictors in the decision-making of adjuvant chemotherapy. No significant interaction was detected among these factors.

**Table 3:** Univariate logistic regression analysis for the use of adjuvant chemotherapy

	Total Distribution	Adjuvant Chemotherapy	%	No Adjuvant Chemotherapy	%	P Value
**Occupation**						
Housewife	173	123	71.1	50	28.9	0.195
Manual Worker	1893	1301	68.8	592	31.2	
Mental Worker	1137	768	67.5	369	32.5	
Others	373	256	68.7	117	31.3	
**Education**						
None	186	122	65.7	64	34.3	0.005
Primary School	462	328	71.0	134	29.0	
Middle School	606	420	69.3	186	30.7	
High School	441	321	72.8	120	27.2	
University and above	396	296	74.8	100	25.2	
**Marital Status**						
Single	51	32	63.6	19	36.4	0.682
Married	4090	2789	68.2	1301	31.8	
Widowed/Divorced	52	35	67.3	17	32.7	
**Age**						
≤ 39 yrs	790	665	84.2	125	15.8	<0.001
40-49 yrs	1624	1330	81.9	294	18.1	
50-59 yrs	1147	912	79.5	235	20.5	
60-69 yrs	483	317	65.6	166	34.4	
≥ 70 yrs	166	47	28.3	119	71.7	
**Stage**						
I	663	458	69.1	205	30.9	<0.001
II	1891	1573	83.2	318	16.8	
III	788	740	93.9	48	6.1	
**ER/PR**						
ER- PR-	1139	972	85.3	167	14.7	<0.001
ER+/PR+	2395	1924	80.3	471	19.7	
**HER2**						
Negative	736	595	80.8	141	19.2	<0.001
Positive	2113	1742	82.4	371	17.6	

#### Stage

The percentage of patients receiving adjuvant chemotherapy in stage I, II and III cases were 69.1%, 83.2% and 93.9%, respectively. In lymph node positive and negative patients, 85.8% (1628/1897) and 74.3% (1475/1984) received adjuvant chemotherapy, respectively. The percentage of adjuvant chemotherapy decreased from 73.7% in 1999 to 60.7% in 2008 in stage I disease, increased from 79.7% in 1999 to 97.1% in 2008 in stage III patients, and remained steady in stage II patients during this 10 years (Table 4).

**Table 4:** Percentage of patients receiving adjuvant chemotherapy according to different age groups, stages, ER/PR and HER2 status from 1999 to 2008

	1999	2000	2001	2002	2003	2004	2005	2006	2007	2008	Total
**Age groups**											
≤39	84.8%	87.3%	85.5%	88.9%	84.5%	87.1%	92.1%	85.3%	74.2%	82.3%	84.2%
40~49	74.7%	82.3%	83.6%	82.4%	85.9%	82.2%	87.5%	81.4%	82.4%	78.2%	81.9%
50~59	76.9%	73.4%	81.8%	80.0%	82.8%	78.5%	77.3%	83.2%	80.1%	78.8%	79.5%
60~69	56.8%	48.7%	56.3%	65.8%	64.2%	76.9%	71.4%	68.5%	67.1%	76.2%	65.6%
≥70	16.7%	25.0%	16.7%	31.3%	17.6%	42.9%	43.8%	28.0%	19.0%	38.1%	28.3%
**Stages**											
I	73.7%	69.0%	66.7%	72.3%	66.7%	75.0%	72.7%	66.7%	64.8%	60.7%	69.1%
II	79.8%	79.1%	79.7%	87.7%	83.4%	86.5%	84.5%	86.3%	82.0%	83.7%	83.2%
III	79.7%	92.1%	94.0%	88.3%	90.3%	100.0%	98.6%	97.7%	99.0%	97.1%	93.9%
**HR status**											
ER+ and/or PR+	86.5%	81.8%	78.7%	83.7%	79.3%	83.3%	80.8%	80.6%	67.8%	76.0%	80.3%
ER- and PR-	82.4%	83.9%	92.7%	88.1%	86.0%	87.6%	92.9%	76.5%	83.9%	81.3%	85.3%
**HER2 status**											
Positive	91.7%	82.4%	83.7%	84.7%	82.4%	83.7%	82.3%	78.9%	78.6%	82.7%	82.4%
Negative	70.3%	83.9%	86.3%	83.3%	83.1%	86.5%	85.4%	83.5%	73.4%	75.8%	80.8%

#### Age

Of the 3271 patients who received adjuvant chemotherapy, 40.6% were diagnosed at the age between 40 and 49 years old, and 28.0% were between 50 and 59. From 1999 to 2008, the percentage of adjuvant chemotherapy in women younger than 60 years remained stable, while significantly increased in women between 60 and 69 and older than 70 years (increased from 56.8% to 76.2% and from 16.7% to 38.1%, respectively; Table 4). Age distribution of patients who received adjuvant chemotherapy was significantly different among different years (χ^2^ = 32.2790, P = 0.0002).

For the entire population (N=4211), the age distribution was significantly different across years (P<0.0001). The percentages of patients in 30-39 and 40-49 age groups were higher in 2008 than in 1999, while the percentage of older patients was lower in 2008 than in 1999.

#### Hormone receptor status

Among 3529 cases with hormone receptor (HR) testing, 2395 (67.9%) patients were HR positive (ER and/or PR positive). In HR positive and HR negative (ER and PR negative) patients, 80.3% and 85.3% received adjuvant chemotherapy, respectively. As illustrated in Table 4, the percentage of HR positive women who received adjuvant chemotherapy decreased from 86.5% in 1999 to 76.0% in 2008, while in HR negative patients, this percentage remained stable.

#### HER2 status

HER2 testing was performed in 56.4% of patients in 1999, which increased to 83.8% in 2008. The southwest (38.3%) and northwest (43.5%) area had less patients tested for HER2 status compared to other regions. The HER2 positive (IHC 3+ or FISH amplified) rate was 25.8% in 2849 patients who were tested for HER2. In HER2 positive and HER2 negative women, 82.4% and 80.8% received adjuvant chemotherapy, respectively. In 736 HER2 positive patients, only 19 (2.6%) received adjuvant trastuzumab therapy, and the majority of them were diagnosed in 2007 (4 cases) and 2008 (10 cases).

### Adjuvant chemotherapy regimens

**Figure 1:**
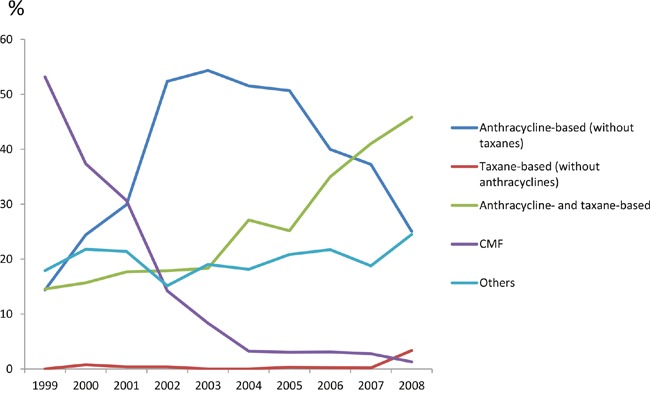
Percentage of adjuvant chemotherapy regimens for breast cancer treatment from 1999 to 2008 (after normalization of age)

Among the 3271 patients who received adjuvant chemotherapy, 1258 (38.5%) received anthracycline-based (without taxanes) regimens, 893 (27.3%) received both anthracycline and taxane, and 449 (13.7%) received CMF. The percentage of CMF decreased significantly during 1999-2008, and regimens containing both anthracyclines and taxanes increased significantly. The percentage of anthracycline-based (without taxanes) regimens increased in the first 5 years and decreased afterwards (Figure 1).

As shown in Supplementary Figure 1, 83.3% of early stage breast cancer in the central area received both anthracyclines and taxanes in adjuvant therapy, which was extremely higher than in other areas (range from 5% to 35%). The percentage of anthracycline-based (without taxanes) regimens was the highest in the east area (54.4%), and was the lowest in the central and southwest areas (12.7% and 23.1%, respectively). Significant differences were observed among 7 traditional regions of China (χ^2^ = 32.2790, P = 0.0002).

**Figure 2:**
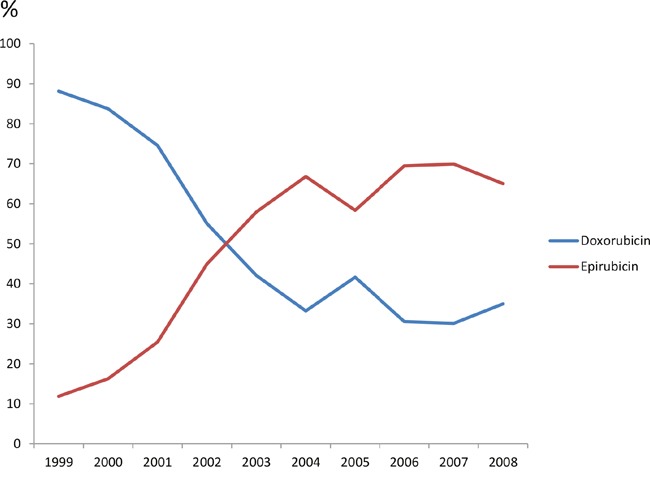
Percentage of doxorubicin and epirubicin in adjuvant chemotherapy regimens containing anthracyclines from 1999 to 2008

Among 2151 patients treated with anthracyclines (with or without taxanes), 959 (44.6%) received doxorubicin, and the rest 55.4% received epirubicin. The percentage of doxorubicin dropped from 92.1% in 1999 to 35.0% in 2008, while the percentage of epirubicin increased from 7.9% to 65.0% (Figure 2). Comparing different regions, patients in the central and northwest areas received more doxorubicin (94.5% and 58.1%, respectively), while more epirubicin was applied in other regions.

Among 912 patients treated with taxanes in adjuvant therapy, 787 (86.3%) received docetaxel, and the rest 13.7% received paclitaxel. Patients in the north area received more paclitaxel (82.0%) than docetaxel, while patients in other regions received more docetaxel.

### Neoadjuvant chemotherapy

A total of 558 (13.3%) patients received neoadjuvant chemotherapy. The percentage of neoadjuvant chemotherapy increased from 10.4% in 1999 to 16.1% in 2008. More clinical T3 and T4 patients received neoadjuvant chemotherapy (17.1% and 24.0%, respectively); 8.2% and 24.1% of lymph node negative and positive patients received neoadjuvant chemotherapy, respectively.

**Figure 3:**
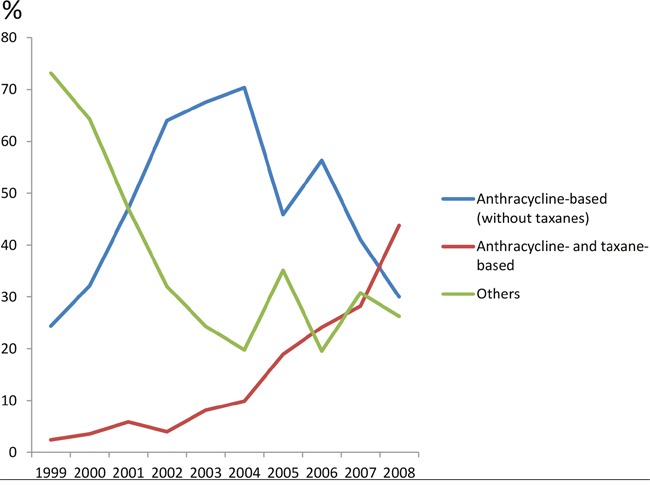
Percentage of neoadjuvant chemotherapy regimens for breast cancer treatment from 1999 to 2008

In analysis of neoadjuvant regimens (n=558), 48.3% were anthracycline-based (without taxanes) regimens, and 18.4% contained both anthracyclines and taxanes. From 1999 to 2008, the percentage of anthracycline-based regimens (without taxanes) increased in the first 5 years and decreased afterwards, while regimens containing both anthracyclines and taxanes increased constantly from 1999 through 2008 (Figure 3). The percentage of other regimens was the highest in 1999 and decreased afterwards. In regional comparison, 72.2% of patients in the central area received both anthracyclines and taxanes, which was uncommon in the southwest (9.2%) and the northeast (9.4%) area (Supplementary Figure 2).

### Metastatic chemotherapy

Among 4211 selected patients, only 392 (9.3%) cases received chemotherapy in metastatic settings, and this percentage remained stable during this period (Table 1). A total of 327 patients received first-line chemotherapy. Among them, 41 (12.5%) and 286 (87.5%) patients received single-agent and combined chemotherapy, respectively. In 117 patients who received second-line chemotherapy, 23 (19.7%) and 94 (80.3%) patients received single-agent and combination chemotherapy, respectively.

The most frequently selected agents in first-line chemotherapy included taxanes (27.2%), anthracyclines (19.7%), platinum (14.9%) and vinorelbine (11.5%). In second-line regimens, taxanes (22.4%) were also the most common choices, followed by platinum (19.2%), capecitabine (15.1%) and vinorelbine (14.2%).

The most common combinations in first-line included anthracyclines plus taxanes (34.9%), taxanes plus platinum (21.7%) and vinorelbine plus platinum (16.5%). In second-line treatment, taxanes and platinum (23.2%) were most common combinations, followed by taxanes plus capecitabine (21.7%) and vinorelbine plus platinum (18.8%). Among 101 HER2 positive MBC patients, only 28 (27.7%) received anti-HER2 treatment. Twenty-two patients received trastuzumab and 6 received lapatinib.

## DISCUSSION

This is the first nation-wide multi-center epidemiologic study of chemotherapy in adjuvant, neoadjuvant and metastatic settings in Chinese breast cancer patients. This study described the transition and characteristics of chemotherapy as well as regional disparities in China from 1999 to 2008.

Major changes in adjuvant chemotherapeutic regimens were observed between 1999 and 2008. CMF regimen decreased rapidly from 1999 to 2004, and the percentage of anthracycline-based regimens increased and become the primary adjuvant regimen from 2001 to 2005. In the meantime, several publications including the EBCTCG meta-analysis demonstrated prognostic benefit of anthracycline-based regimens over CMF [7, 8]. After 2006, regimens containing both anthracyclines and taxanes became the mainstream. Simultaneously, multiple studies suggested that addition of taxanes was associated with more survival benefit compared to anthracyclines alone, especially in high-risk women [9].

This transition of adjuvant chemotherapy in China (Figure 1) was 2 to 3 years later than that in other countries [10–12]. Based on contemporary observations from North America and Europe, regimens containing both anthracyclines and taxanes became the main choice since 2004 [10–12]. This delay was probably due to delayed entrance of taxanes into the drug reimbursement list in China [13].

Our analysis reported the transition from doxorubicin to epirubicin between 1999 and 2008, which reflected a better understanding of the differences between toxicity profiles, especially cardiotoxicities of these two drugs in China [14, 15].

Several independent factors were detected to affect the application of adjuvant chemotherapy. Similar to other studies [16, 17], primary tumor stages were associated with adjuvant chemotherapy application. The percentage of adjuvant chemotherapy in stage I patients decreased from 1999 to 2008, and increased in stage III cases. [18] This difference between stage I and stage III women in China reflected a transition from uniform treatment to differentiated and risk-adapted adjuvant chemotherapy based on evidence and international recommendations [18].

The percentage of early-stage patients who received adjuvant chemotherapy in our study was higher than western countries [11, 17, 19]. This difference could be explained by an earlier onset of breast cancer in China than in western countries. The median age at diagnosis was around 45 to 49 [20–22]. The percentage of patients younger than 35 years was higher in China [20–23]. Younger patients have better performance status and tolerance.

In our analysis, the percentage of HR positive women who received chemotherapy decreased during this 10-year period. This transition reflected the evidence [24–26] that adjuvant chemotherapy in HR positive patients was associated with smaller survival benefit than in HR negative patients.

The majority (87.5%) of metastatic patients received combination regimens as first-line chemotherapy, which was significantly higher than in other countries [12, 27, 28]. This preference for combination chemotherapy reflected the desire for rapid disease remission and better performance status and tolerance of Chinese breast cancer patients.

In addition to anthracyclines plus taxanes, platinum-based doublets were also frequently selected, such as taxanes plus platinum or gemcitabine plus platinum. Platinum was less expensive than many other agents and had synergistic activity with several agents [29–32]. Capecitabine was more common in second and third lines.

Enormous geographical differences were detected (Table 2). One possible reason would be socioeconomic discrepancies. The percentage of chemotherapy was higher in more prosperous eastern, southern and northern areas than other parts of China. The other reason would be diverse local health insurance policy. Paclitaxel, but not docetaxel, was covered by medical insurance in EBC patients in the north area, which greatly affected the selection of taxanes.

However, our analysis has several potential limitations. Firstly, selection bias might exist as no less elite hospitals were selected from the same area. Secondly, data quality was largely dependent on the thoroughness and accuracy of documentation of medical history and treatment. Thirdly, specific schedules, doses and side effects of certain chemotherapy regimens were not designed to be collected in this study. Finally, as an epidemiologic study, no survival follow-up was performed.

In conclusion, our study presented detailed information on characteristics and trends of chemotherapy regimens in Chinese breast cancer patients between 1999 and 2008. Stage, age, HR and HER2 status were all independent factors affecting decisions of chemotherapy in EBC women. The percentage of combination chemotherapy in advanced stage patients was higher than in Europe and North America. In addition, key findings from large clinical trials were incorporated into practice, but due to late access to new drugs and limitations of reimbursement policies, the evolution of regimens and agents were slower in China. Substantial geographical disparities could be attributed to regional socioeconomic inequalities and different health insurance policies.

## MATERIALS AND METHODS

### Study design

This study was a nation-wide multi-center retrospective epidemiologic study of randomly selected breast cancer patients over a 10-year interval in China. This study was approved by the Cancer Foundation of China Institutional Review Board.

### Hospital selection

The hospital selection and case sampling methods have been previously described in detail [20]. According to traditional administrative district definition, China was stratified into 7 geographic regions (north, east, south, northeast, northwest, central and southwest). Convenience sampling was used to select one tertiary hospital from every region on the basis that (1) it was one of the best leading tertiary hospitals and had regional referral centers providing pathology diagnosis, surgery, radiotherapy, medical oncology, and routine follow-up care for patients with breast cancer; (2) inpatients were from all over the region; and (3) breast cancer screening practices were in accordance with Chinese national standards.

### Patients

Female primary breast cancer inpatients in one randomly selected month each from year 1999 to 2008 were enrolled in this study. January and February were excluded due to Chinese annual holiday (the Spring Festival) [20]. In order to avoid selection bias, an enrolment scheme was used. Inpatients from alternating prespecified month of each year were enrolled. For example, in the year of 1999, pathology confirmed breast cancer patients admitted in March would be enrolled; in the year of 2000, patients admitted in Apirl would be enrolled. Beside, all patients in one selected month were reviewed. If inpatients in one selected month were less than 50 in that year, more cases from neighbor months were included until it reached 50 in total. To ensure this study to be geographically representative, we included patients from hospitals of all 7 regions across China.

All patients enrolled were required to meet the following inclusion criteria [20]: (1) pathologically confirmed primary breast cancer; (2) admission date was within the selected month in each hospital and (3) received treatment (surgery, medical therapy and/or radiotherapy) against breast cancer. Pathologic diagnosis of patients was based on the 1981 and 2003 WHO histological classification criteria [33, 34] and the 1997 and 2002 AJCC TNM staging systems [35, 36].

### Data collection and quality control

As described previously [20], the following data were collected for all selected patients via medical chart review: (1) general information including date of diagnosis, inpatient admission date, diagnosis at admission, inpatient discharge date, and discharge outcome; (2) demographic characteristics including age, occupation, height, weight, education and marital status; (3) results of the clinical breast examination (CBE) and diagnostic imaging; (4) use of currently available surgery approaches, radiotherapy, chemotherapy and targeted therapy; (5) pathological characteristics including preoperative cytology and pathologic examinations, postoperative pathology, estrogen and progesterone receptor expressions and human epidermal growth factor receptor2 (HER-2) expression. All above information was recorded in the designed case report form (CRF) and was inspected for consistency [20].

### Statistical analysis

Study participants were grouped according to 7 categories of regions (North, North-East, Central, South, East, North-west, and South-west) or year of diagnosis. We used the logistic regression model to examine the relations between adjuvant chemotherapy and its possible predictors. Tests for trend across the year of diagnosis for percentage of participants were performed using Cochran-Armitage Test. All P values were two-sided and P values less than 0.05 were considered statistically significant. Analyses were conducted using SAS version 9.1.3 service package 4 (SAS Institute Inc, Cary, NC).

## SUPPLEMENTARY MATERIALS AND FIGURES


