# Supplementary material for: A nation-wide multicenter 10-year (1999–2008) retrospective study of chemotherapy in Chinese breast cancer patients
Source: Oncotarget. 2017 Mar 22;8(44):75864–73. doi: 10.18632/oncotarget.16439 (PMC5652669; doi:10.18632/oncotarget.16439)
Supplement: Supplementary file 1 [file oncotarget-08-75864-s001.pdf]

A nation-wide multicenter 10-year (1999–2008) retrospective study of chemotherapy in Chinese breast cancer patients

SUPPLEMENTARY FIGURES

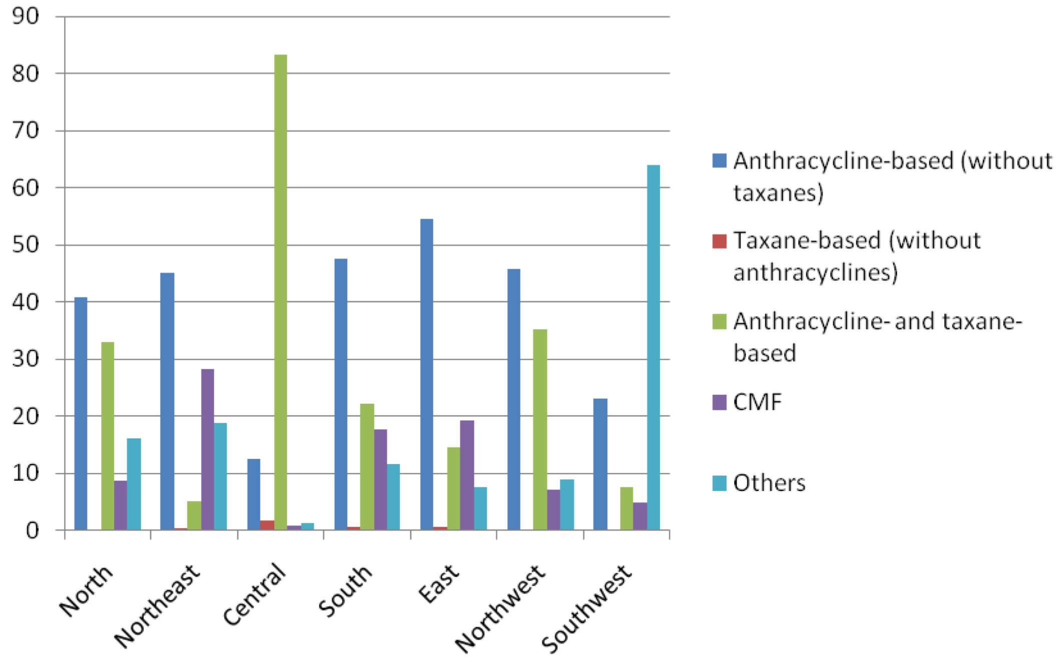

Supplementary Figure 1: Percentage of adjuvant chemotherapy regimens for breast cancer treatment in 7 geographic regions of China.

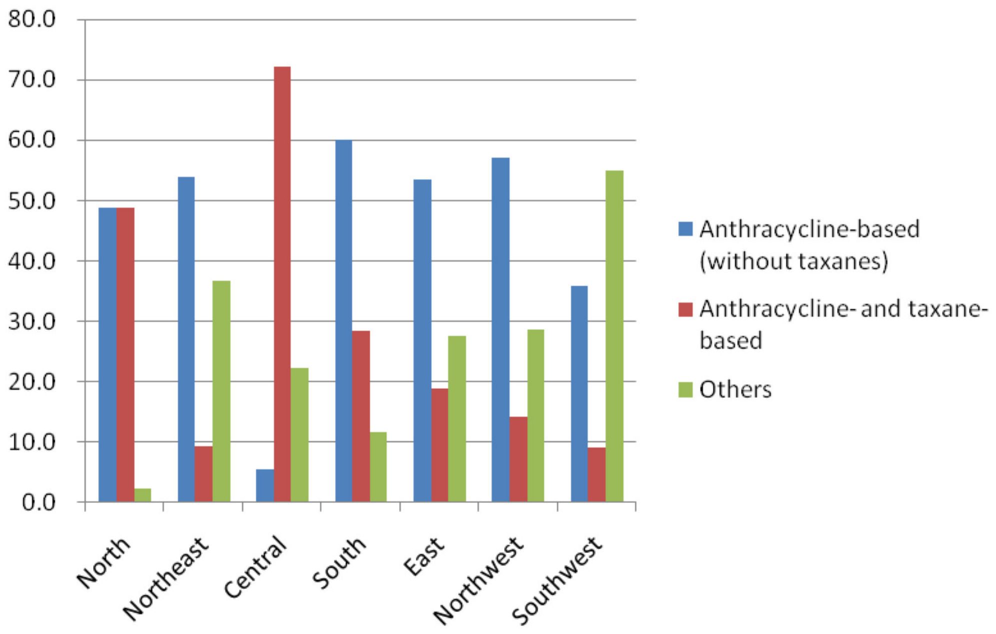

Supplementary Figure 2: Percentage of neoadjuvant chemotherapy regimens for breast cancer treatment in 7 geographic regions of China.
